# Synergistic Effect of Barbadensis miller and Marsdenia Condurango Extracts Induces Apoptosis Promotes Oxidative Stress by Limiting Proliferation of Cervical Cancer and Liver Cancer Cells 

**DOI:** 10.31557/APJCP.2021.22.3.843

**Published:** 2021-03

**Authors:** Tahir Maqbool, Faheem Hadi, Sehrish Razzaq, Sadia Naz, Saira Aftab, Sameera Khurshid, Sana Javaid Awan, Aisha Nawaz, Farah Abid, Arif Malik

**Affiliations:** *Faculty of Pharmacy, University of Lahore, Defence Road, Lahore, Pakistan. *

**Keywords:** Synergistic effect, cytotoxicity, protein expression, gene expression

## Abstract

**Background:**

Drug synergy is the combine effect of drug efficacy. Synergistic combinations of active ingredients have proven to be highly effective and more useful in therapeutics. In contrast, the individual effect of drug is usually undesirable and mostly used for selecting drug-resistant mutations. Purpose of this study was to check synergistic effects of both plants (Barbadensis miller and Marsdenia condurango) against liver and cervical cancer.

**Methodology:**

Culturing of HeLa (cervical cancer cell line) and HepG2 (liver cancer cell line) cells, IC_50_ evaluation, viability assays (trypan blue, crystal violet), p53 ELISA and immunocytochemistry, MUSE analysis (count and viability), antioxidants (GSH, SOD, CAT), at the end RT-PCR was performed.

**Results:**

IC_50_ evaluation was done of each plant individually and with combination for synergistic effects, IC_50_ with plants combination (synergism) was applied on further viability assays (trypan blue, crystal violet, MUSE analysis via count and viability kit) p53 ELISA and immunocytochemistry for evaluation of cellular apoptosis, antioxidants assays (GSH, SOD, CAT), and RT-PCR with proliferative and apoptotic markers along with internal control.

**Conclusion:**

According to current study it was observed that synergistic effect of these plants has more anticancer properties with minimum effective dose. It was also observed that extracts possess the ability to induce apoptosis, restrict proliferation and enhanced oxidative stress.

## Introduction

Many people have envisioned drug combinations as promising methods of treating complex diseases such as inflammation, cancer and type 2 diabetes (Feala et al., 2010; Fitzgerald et al., 2006; Keith et al., 2005). However, when combined, the drugs interact in many unexpected ways they show a variety of different results (Yeh et al., 2009). Among these interactions, drug synergy and antagonism have received particular attention. Drug synergy is the combined effect of drug (Fitzgerald et al., 2006). Synergistic combinations of active ingredients have proven to be highly effective and more specific as treatment therapies (Lehár et al., 2009). In contrast, drug antagonism is usually undesirable, but can be useful in selecting drug-resistant mutations (Chait et al., 2007). Drug interactions can also be determined in this way, so that the biological network structure of the drug target under study can clarify the mode of action of the drug (Araujo et al., 2007; Yang et al., 2008; Yang et al., 2007) and interact (Lehár et al., 2007; Yeh et al., 2006). Indeed, an early study theoretically proved that continuous inhibition of the enzyme chain can lead to drug synergy (Black, 1963), mentioned by Fitzgerald et al (Fitzgerald et al., 2006). Different modes of collaborative combination have been studied in several typical network environments as mentioned by Lehar et al (Lehár et al., 2007). Antitumor and cytotoxic potential of Barbadensis miller extract (BE) continues to be the center of interest for scientific research since 1980’s and has extensively been used as an anticancer potential drug (Candoken et al., 2017) Marsdenia cundurango (Condurango), commonly known as condor vine. It is a medicinal plant whose bark extract (“*mother tincture*”) is traditionally used in homeopathic medicines. It is used as a therapeutic agent for stomach cancer. Condurango extract (CE) is also used as an appetite stimulant and has relatively few side effects when taken at the recommended dose. In addition, CE has been shown to have anti-inflammatory effects in animal studies (Bishayee et al., 2015).

Hepatocellular carcinoma (HCC) is the most common form of liver cancer and is often associated with a very poor prognosis (Thorgeirsson and Grisham, 2002) and the third most common cause of death worldwide. Mostly, HCC is associated with severe liver fibrosis and cirrhosis caused by chronic liver inflammation. Therefore, the risk of liver cancer depends on the background liver factor, where fibrosis is the main determinant. (Ghany et al., 2003), this is due to the accumulation of reactive oxygen species (ROS) in damaged hepatocytes, an increase in oxidative stress in hepatocytes may explain the close relationship between liver fibrosis and hepato-carcinogenesis (Sakurai et al., 2013). 

Cervical cancer is the second most common malignancy among women worldwide (Gao et al., 2013). It is the commonest malignancy of the female genital tract in developing countries (Cronje 2004; Uzoigwe and Seleye-Fubara, 2004). It has a heavy global burden, with 530,000 new cases and 275,000 maternal deaths reported annually (Goddy et al., 2015). Approximately 83% of these cases occur in developing countries, where it is the leading cause of cancer-related death among women.

## Materials and Methods

*Plant preparation*

Barbadensis miller (whole plant) and Marsdenia cundurango (bark) preparation was done according to Abid F protocol (Abid et al., 2020) protocol. The final extract was dissolved in DMSO in different concentrations according to need.

*Culturing of HeLa and HepG2 Cell Lines*

HeLa cell line was obtained from American Type Culture Collection (ATCC®). Culture was maintained at 37ºC and 5% CO_2_ with 95% humidity level. Complete culturing medium contained 10% FBS, 1X Penicillin/Streptomycin solution (Thermo Scientific Cat#15140122) in DMEM high glucose (Thermo Scientific Cat#12800017). Culture was expanded and stored both in -80 freezers and liquid nitrogen containers as well and taken out when needed. 

*Treatment of HeLa and HepG2 cell lines with plant extracts*

HeLa and HepG2 cells were cultured onto 96-well plate for IC_50_ calculation. Cultured HeLa and HepG2 cells were divided into seven groups in case of each plant while it was further reduced to five groups in case of synergistic effect of combined plant extracts for each cell line for IC_50_, In case of HeLa, ethanolic extract of Barbadensis miller (BE), UT is untreated group, B-H (10, 25, 50, 100, 500, 1000 µg/ml) was treated HeLa group, while in case of HepG2, UT is untreated group, B-HG (10, 25, 50, 100, 500, 1,000 µg/ml) was treated HepG2 group. In case of HeLa, ethanolic extracts of Condurango (CE), UT is untreated group, C-H (10, 25, 50, 100, 500, 1,000 µg/ml) was treated HeLa group, while in case of HepG2, UT is untreated group, C-HG (10, 25, 50, 100, 500, 1,000 µg/ml) was treated HepG2 cells, In case of synergistic effects on HeLa, combination of ethanol extracts, UT is untreated and S-H (10+10, 25+25, 50+50, 100+100 µg/ml) was treated HeLa group and for HepG2 UT is untreated, S-HG (10+10, 25+25, 50+50, 100+100 µg/ml) was treated HepG2 group. After that IC50 of combined plant extracts, cells were divided into three groups in case of each cell line for analysis of further assays, viability assays (crystal violet assay, trypan blue assay), ELISA p53, MUSE analysis, antioxidant assay and Lactate dehydrogenase assay (LDH) were performed in 96 well plate while immunocytochemistry p53 was performed in 24 well plate.

*IC*_50_ *Evaluation*

IC_50_ was evaluated via 3-(4,5-dimethylthiazol-2-yl)-2,5-diphenyltetrazolium bromide (MTT) assay after 72 hrs treatment. Monolayer of cells was first washed with phosphate buffer saline (PBS) (Invitrogen Inc., USA), further cells were incubated in 100 µl complete medium containing 25 µl MTT solution (Invitrogen Inc., USA) for 2 hrs. MTT converted into purple colored formazan in living cells which was then solubilized with dimethyl sulphoxide (DMSO) (Invitrogen Inc., USA) and absorbance of solution was taken at 570 nm.

*Trypan Blue Assay*

Trypan blue assay was performed according to maqbool T protocol (Maqbool et al., 2019).

*Crystal Violet Assay*

Cell viability was also assessed by crystal violet staining method. This method was conducted in a 96-well plate accoding to maqbool T protocol (Maqbool et al., 2019)

*Enzyme linked immunosorbent assay (ELISA)*

Solid phase sandwich ELISA was performed for p53 (Santa Cruz Biotechnology, USA) in a 96-well plate (Corning, USA). Performed according to (Abid et al., 2020) protocol absorbance was taken at 450 nm by using the microtiter plate.

*Immunostaining*

Cells of experimental groups of HeLa and HepG2 cell lines, were plated in different wells of a 6-well plate for characterization of immunostaining following 72 hrs treatment according to (Abid et al., 2020) protocol.

*Muse analysis via count and viability kit*

Ethanolic extracts in combination were applied on HeLa and HepG2 cells which were cultured in 6 well plate through count and viability kit (Cat. No MCH100102) by using the Muse™ automated cell analyzer (Merck-Millipore). Cells after treatment were centrifuged at 2000 RPM for 5 min, supernatant was discarded pellet was dissolved in cell and viability reagent and were counted via MUSE analyzer.

*Evaluation of antioxidative enzymes Glutathione reductase (GSH), Catalase (CAT) assay, Superoxide dismutase (SOD) assays*

GSH, SOD, CAT were performed according to protocol (Abid et al., 2020).

*Estimation of gene expression profiling*

*Gene expression profiling*

After carefully analyzing all the previous experimental results, the most appropriate IC50 concentrations were applied on cell line groups. Total RNA was extracted from cancer cells HeLa, HepG2 cells of all experimental groups by using TRIZOL reagent (Invitrogen, USA). cDNA synthesis was done by Reverse Transcription System (Fermentas, USA) according to the manufacturer’s protocol. Gene expression analysis was carried out by RT-PCR using SYBR Green PCR SuperMix (Fermentas) on BioRad System iQ5. For in-vitro experiments, expression of BAX, PCNA, p53 and Ki67 was estimated. GAPDH was used as an internal control.

*Statistical analysis*

All data of experimental groups was expressed as mean ± SEM in triplicate experiments. For statistical analysis, group means were compared by one-way ANOVA and Bonferroni’s test was used to identify differences between groups. Quantitative data obtained from different experimental groups was statistically analyzed via graph pad software by using two ways ANOVA. P value less than 0.05 was considered as significant from statistical analysis. Endnote was used to insert reference.

## Results

*MTT Assay for IC*_50_ *Evaluation*

**Figure 1:**
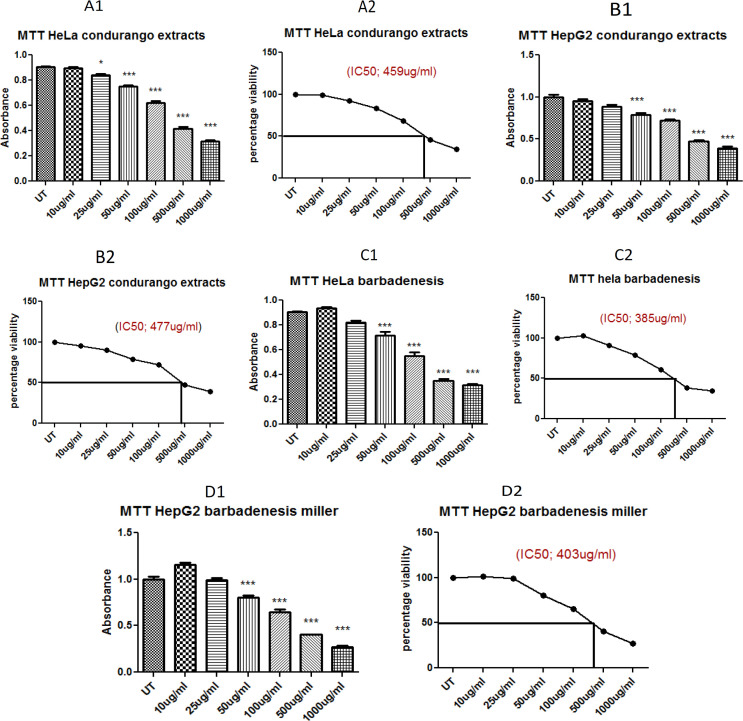
Figure Shows the IC_50_ of *Barbadenesis miller (*BE) and Marsdenia cundurango (CE).

IC50 of ethanolic extracts of Barbadensis miller (BE) and Marsdenia cundurango (CE) extracts separately and combined plant extracts. Figure 1 and Table 1 displayed MTT assay results which is a reliable method for measuring cell viability. Cell viability of plants extracts on HeLa and HepG2 cell line is expressed as percentage cell viability.

IC_50_ calculation was done against HeLa and HepG2 liver cancer cells with increasing concentrations. The IC_50_ value was found to be 403 µg/mL in case of ethanolic extracts of Barbadensis miller and 477 µg/mL in case of ethanolic extracts of Marsdenia cundurango in case of HepG2 cells, and 385 µg/mL in case of ethanolic extracts of Barbadensis miller and 459 µg/mL in case of Marsdenia cundurango in case of HeLa cells.

While in case of combined cytotoxic effect of plants on HepG2 cells was 53µg/mL (1:1 of each plant) and on HeLa cells 49.9 µg/mL (1:1 of each plant).

*Trypan blue cell viability assay*

**Figure 2:**
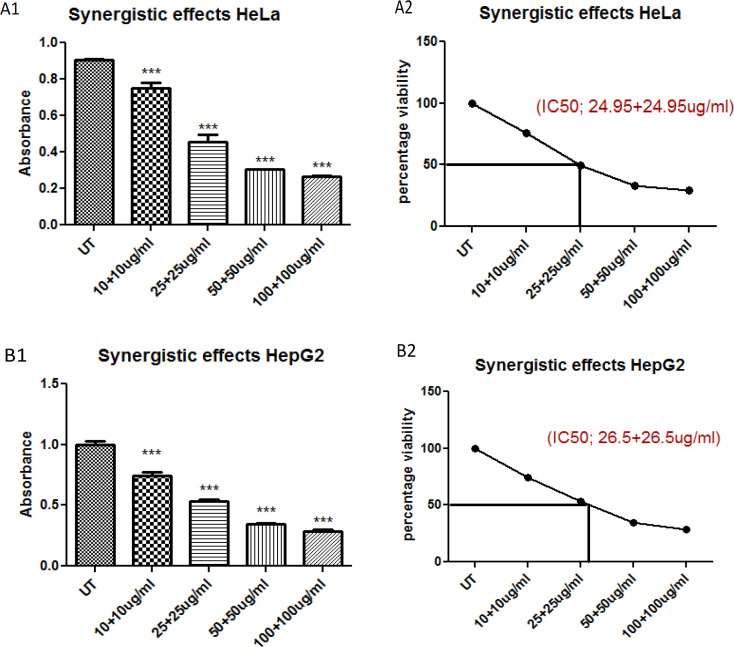
Figure Shows the IC_50_ of Combined/Synergistic BE+CE Extracts

Viability of cells was further assessed by trypan blue cell viability assay for detection and evaluation of dead and live cells as shown in Figure 2 and Table 2. 

HeLa and HepG2 cancer cells were treated with ethanolic extracts via staining with trypan blue. A significantly large number of blue colored cells were observed in treated HeLa and HepG2 cells indicating more dead cells as compared to untreated cells.

*Crystal violet cell viability assay*

Cell viability was also estimated using crystal violet staining of HeLa and HepG2 cell line as displayed in Figure 2 and Table 2. When these cancer cells were treated with combination of BE (ethanolic extracts of Barbadensis miller) and CE (ethanolic extracts of Marsdenia cundurango) extracts, they showed fewer living cells as compared to untreated HeLa and HepG2 cells based upon absorbance. 

*Muse analysis via count and viability kit*

**Figure 3:**
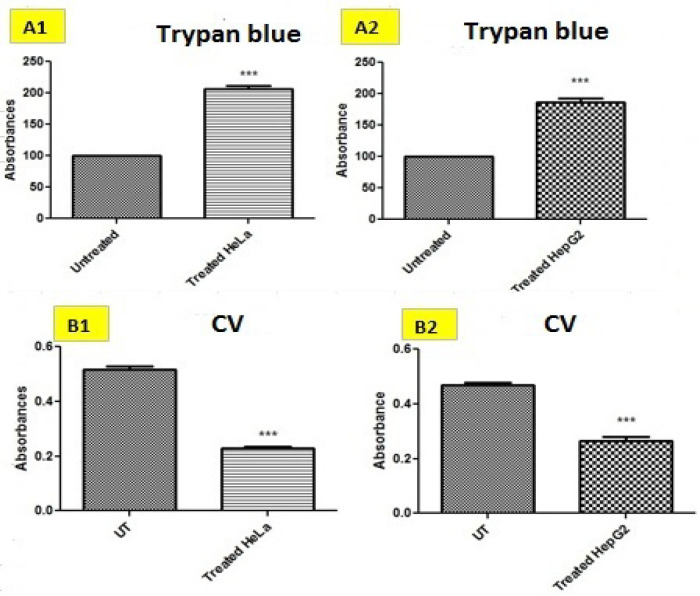
Figure Shows the Combined/Synergistic Effects of Ethanolic Extract of *Barbadensis miller* (BE) and *Marsdenia Cundurango* (CE) in Trypan Blue Assay, Crystal Violet against HeLa and HepG2 Cells

The results of cell count and viability are shown in Figure 3 where cells were treated with ethanolic extract of plants that showed toxicity. HeLa and HepG2 cells treated with combination of BE and CE extracts killed more cells as compared with untreated group and less live cells were observed in treated groups as compared with untreated ones. In case of untreated group HeLa, 80.3% were viable cells, whereas treatment with combined ethanolic plant extract represented 35.4 cells. In case of untreated group HepG2, 81.2% were viable cells, whereas treatment with combined ethanolic plant extract represented 32.0 cells.

*High apoptosis level in post treated HeLa and HepG2 cells via ELISA*

**Figure 4:**
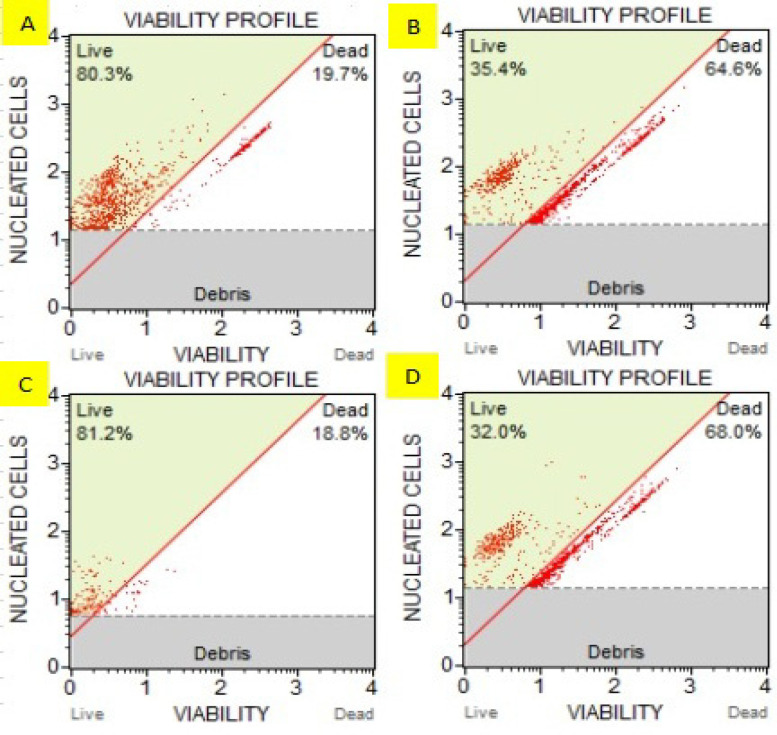
Muse Analysis of HeLa and HepG2 Cells

After treating HeLa and HepG2 cells with combined ethanolic extracts of Barbadensis miller and Marsdenia cundurango, more apoptosis was observed via p53 ELISA shown in Figure 4 and Table 2. According to current results, treatment of cell lines with ethanolic extracts increased the apoptosis level in cancer cells. As p53 is a principal factor of apoptosis, its level was increased in cell line treated with ethanolic extracts.

*Higher expression of apoptosis in post treated HeLa and HepG2 cells via immunocytochemistry*

**Figure 5:**
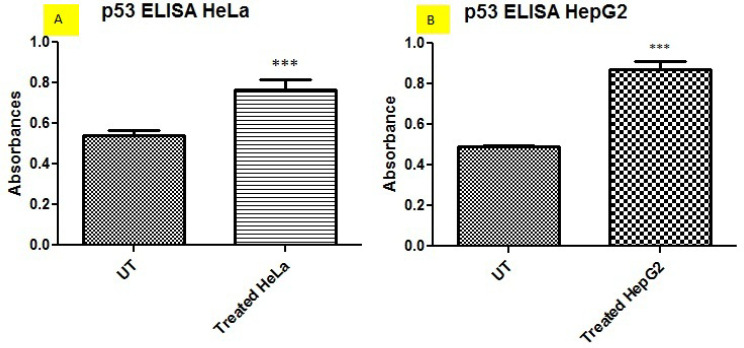
Characterization of p53 Level in Untreated and plants extracts treated HeLa and HepG2 Cells in Synergism via ELISA

After treating HeLa and HepG2 cells with ethanolic extracts of Barbadensis miller Marsdenia cundurango extracts, more apoptosis was seen via p53 immunocytochemistry shown in Figure 5. According to current results, treatment of cell lines with ethanolic extracts enhanced the apoptotic expression of cells in cancer cells. As p53 is a principal factor of apoptosis, its level was increased in cell line treated with ethanolic extracts.

*Decreased antioxidants level in treated HepG2 and HeLa groups*

**Figure 6:**
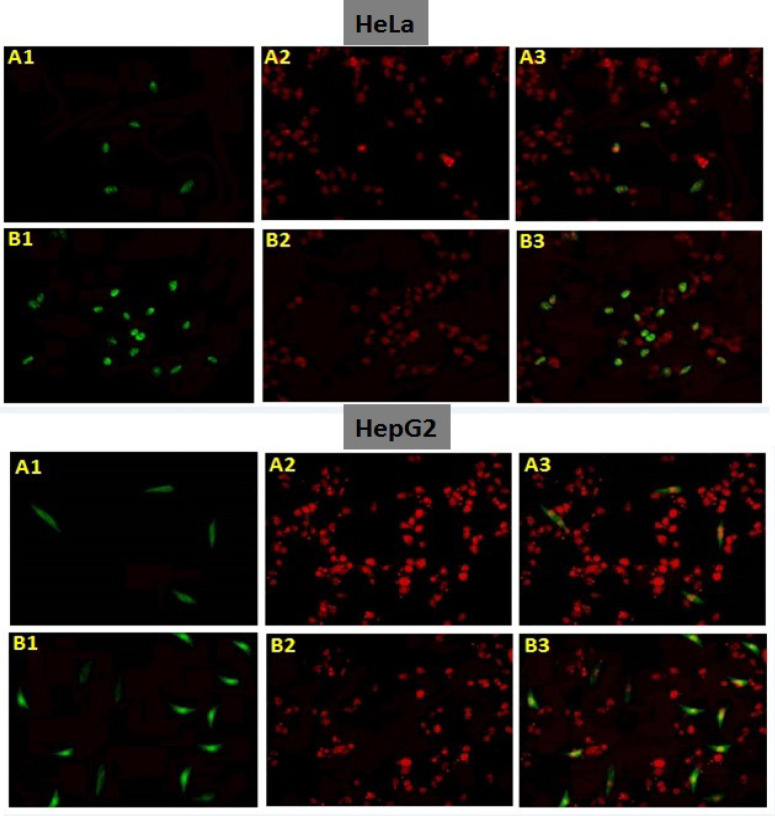
Expression of p53 in Untreated and plants extracts treated HeLa and HepG2 Cells in Synergism via Immunocytochemistry

The antioxidant potential using the catalase (CAT), superoxide dismutase (SOD) and glutathione reductase (GSH) assays which determines the free radical scavenging activity. It was observed that GSH, CAT and SOD activities were decreased in treated groups as compared to untreated groups as reflected in Figure 6 and Table 3.

*Gene expression profiling*

To check the effect of combined plant extracts at the molecular level of cell proliferation and cell apoptosis, cellular proliferation markers (PCNA and Ki67) and apoptosis markers (p53, BAX) levels were assessed via RT-PCR analyses. The results showed that level of proliferative markers was downregulated in treated groups of HeLa and HepG2 cell lines as compared to untreated groups while the level of apoptotic genes was upregulated in treated HeLa and HepG2 cell lines as compared to untreated. In both apoptosis and proliferation, GAPDH served as an internal control as the expression levels of each gene were normalized by GAPDH as shown in Figure 8.

**Table 1:** IC_50_ of BE Extract and CE and Combined BE+CE (Synergistic Effects) Extracts

Cell Line		Absorbance at 570nm for different samples						
		UT	10µg/mL	25µg/mL	50µg/mL	100µg/mL	500 µg/mL	1000 µg/mL
HeLa	BE	0.907±0.0115	0.897±0.0153	0.840±0.0173	0.753±0.0153	0.620±0.0265	0.417±0.0289	0.317±0.0153
	CE	0.907±0.0115	0.933±0.0208	0.823±0.0252	0.717±0.0473	0.550±0.0500	0.350±0.030	0.317±0.0153
	BE+CE (1:1)	0.907±0.0115	0.753±0.0451	0.453±0.0757	0.303±0.0057	0.267±0.0115	--	--
Hep G2	BE	1.00±0.0500	0.957± 0.0404	0.890±0.0361	0.790±0.0361	0.720±0.0265	0.477±0.0252	0.390±0.0361
	CE	1.00±0.0500	1.16±0.0404	0.990±0.0361	0.803±0.0351	0.650±0.0500	0.403±0.00577	0.271±0.0258
	BE+CE combined (1:1)	1.000±0.0500	0.740±0.0529	0.530±0.0265	0.343±0.0153	0.283±0.0289	--	--

**Table 2:** IC_50_ of Combined BE+CE extracts for Trypan Blue, Crystal Violet and ELISA p53

	Mode of measurement		HeLa	HepG2
TB	Relative percentage of live cells	Un treated	100±0.000	100±0.000
		Treated with IC_50_	187±11.5	207±7.77
CV	Absorbance taken at 570 nm	Un treated	0.517±0.0207	0.4673±0.02022
		Treated with IC_50_	0.228±0.014	0.2633±0.2893
ELISA p53	Absorbance taken at 450 nm	Un treated	0.540±0.0482	0.233±0.0257
		Treated with IC_50_	0.493±0.00781	0.268±0.0294

**Table 3:** IC_50_ of Combined BE+CE Extracts GSH, SOD, CAT and LDH

Assay	Mean + SD values	HeLa	HepG2
GSH	Untreated	0.517±0.0351	0.5039±0.03988
	BE+CE	0.298±0.0107	0.3281±0.01893
SOD	Untreated	0.561±0.0321	0.5613±0.05552
	BE+CE	0.336±0.0222	0.3682±0.01900
CAT	Untreated	0.517±0.0207	0.6068±0.02305
	BE+CE	0.302±0.00650	0.3412±0.01000

**Figure 7:**
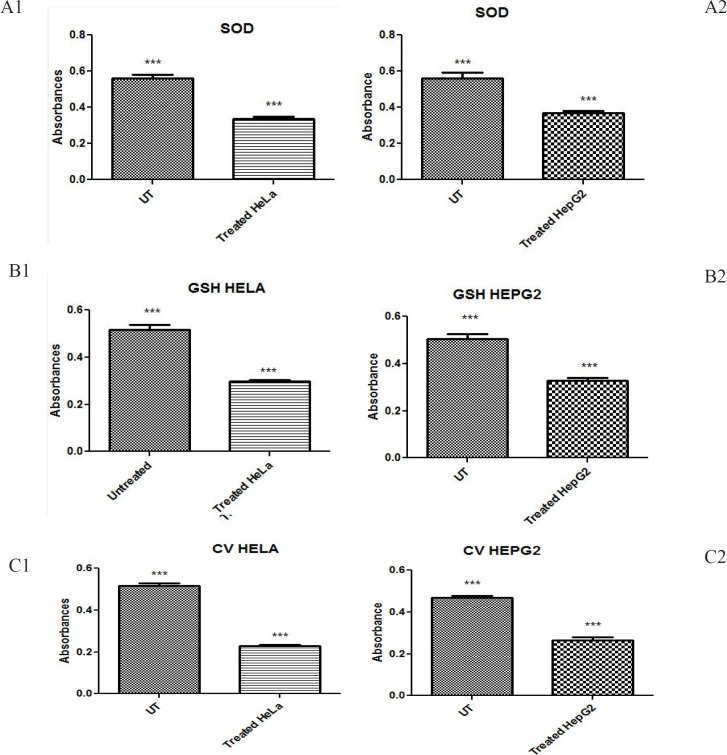
Shows Antioxidant Assays (SOD, CAT, GSH)

**Figure 8:**
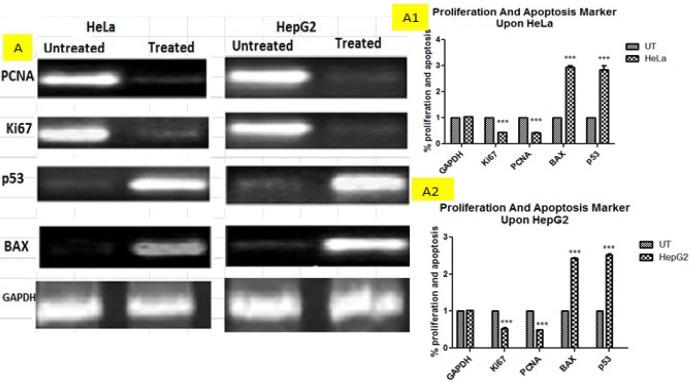
Figure Shows the Relative Gene Expression

## Discussion

Multidrug therapy is a helpful strategy focused on the direct blocking or killing of damaging agents (such as cancer cells or pathogens) as well as on the activation of human body defenses or repair mechanisms. It derives from a gradual withdrawal of the previously adopted dogma of mono-drug therapy; for decades, pharmacological research was based on the identification of a single active principle (Doos et al., 2014). As regards phytotherapy research, only recently have the contributions of traditional Chinese medicine, Ayurveda and traditional Western phytomedicines begun to be scientifically confirmed and appreciated. Furthermore, during the last 20 years, the world has encountered an increasing rate of the use of conventional medicines combined with complementary and alternative medicine (CAM), which are represented not only by homeopathy, naturopathy, chiropractic, and energy medicine, among others, but also ethnopharmacology and phytotherapy (Obodozie, 2012). It is becoming evident that many diseases have a multifaceted etiology, which could be treated more successfully with a drug combination strategy than a single administration. In western countries, for multifactorial or complex disease treatment (Katselou et al., 2014; Mohammad et al., 2006; Papazisis et al., 2006; Spagnuolo et al., 2015; Chen et al., 2016; Salehi et al., 2019) in the present study activity of both plants were individually assessed using only MTT while combined plant extracts were analyzed using MTT and rest of assays are described below. 

Cytotoxicity assay that depend on the conversion of substrate to chromogenic product by live cells, the MTT assay is still among one of the most versatile and popular assays. The MTT assay involves the conversion of the water-soluble yellow dye MTT [3-(4,5-dimethylthiazol-2-yl)-2,5-diphenyltetrazolium bromide] to an insoluble purple formazan by the action of mitochondrial reductase (Kumar et al. 2018). Activity of ethanolic extracts of both plants Barbadensis miller (BE) and Marsdenia cundurango (CE) were assessed by MTT via application of different doses i.e., 10, 25, 50, 100, 500 and 1,000 µg/mL where BE and CE showed higher IC50 on HeLa and HepG2 which was 403 and 385 µg/mL and 477 and 459 µg/mL respectively while in case of synergistic effect IC_50_ was very less which is a great sign as an anticancer agent against HeLa and HepG2 cell line which was 49.90 µg/mL (1:1 of each plant) on HeLa and 53 µg/mL (1:1 of each plant) on HepG2 (Figure 1). According to finding combined plant extracts (synergistic effects) have potential anticancer activity at very less dose. 

Moreover, trypan blue exclusion test is used to determine the number of viable cells present in a cell suspension. It is based on the principle that live cells possess intact cell membranes that exclude certain dyes, such as trypan blue, eosin, or propidium, whereas dead cells do not. In this test, a cell suspension is simply mixed with dye and then visually examined to determine whether cells take up or exclude dye (Strober, 2015). In current study, evaluation of cell viability by the trypan blue method showed an increased trend against IC_50_ of combined plant extracts of BE+CE. The increased percentage of dead cells suggesting that disrupted cell membrane of hepatocellular carcinoma and cervical cancer and is very effective against hepatocellular carcinoma (Figure 2). Furthermore, crystal violet staining is a quick and versatile assay for screening cell viability under diverse stimulation conditions (Geserick et al., 2009). However, it is potentially compromised by proliferative responses that occur at the same time as cell death responses. Therefore, chemical inhibitors of caspases and/or of necroptosis may be incorporated into the assay (Degterev et al., 2008; Sun et al., 2012). Alternatively, molecular studies (e.g., overexpression or knockdown) can be performed to address the nature of cell death more specifically (Feoktistova et al., 2011). Evaluation of cell viability by the crystal violet method showed a decreasing trend against IC_50_ of BE+CE. The decreased percentage of viable cells suggesting that plant combination treatment is very effective against hepatocellular carcinoma and cervical cancer as there are less viable cells in treatment group as compared with untreated (Figure 2).

Count and viability assay are a fluorescent-based analysis using flow cytometry, which is more specific and reliable to quantify the number of viable cells. The DNA binding dyes present in the reagent differentially stain viable and non-viable cells based on their permeability and provide an accurate count of both the cells (Jose et al., 2018). Figure 3 indicates the less live cells and more dead cells in case of treatment with combined plant extracts as compared with untreated group where more live cells and fewer dead cells were observed.

Apoptosis induction is a useful strategy for anticancer drug development. Plant derived anti-cancer drugs exert cell death by inducing apoptosis in cancer cells. Many mechanisms responsible for apoptosis induced by plants and most of them induce apoptotic cell death by intrinsic or extrinsic pathway and p53 dependent or independent pathway, in our finding it was observed that plant extracts induced apoptosis in HepG2 and HeLa cells via p53 ELISA and Immunocytochemistry (Figures 4 and 5). Also our results showed that the cytotoxic effect of combined plant extracts is due to its potential to induce apoptosis by the upregulation of apoptotic markers (p53, BAX) and reduction of proliferation by the downregulation of proliferative genes (PCNA, Ki67) in HeLa and HepG2 cells (Figure 8).

In this study we also did the antioxidant evaluation of plants by SOD, catalase and GSH, as oxidative stress is a primary marker for cancer (Bailey et al., 2012; Lendahl et al., 2009; Toyokuni, 2008). Anti-oxidative enzymes effect the proliferation of cells in positive way. But when antioxidants are given with Anti-proliferative therapy it will enhance the effect of therapy and improve anticancer effect. The SOD, catalase and GSH activities were increased when treated with plant extracts (Figure 6).

In conclusion, this study was based upon synergistic effects of ethanolic extracts of Barbadensis miller and Marsdenia cundurango upon HeLa and HepG2 cells. It was observed that combined plant extracts have more anticancer potential with low dose as compared with individual plant extracts. According to our finding it was observed that combined plant extract induce apoptosis, restrict proliferation and enhanced oxidative stress.

## Author Contribution Statement

This study was designed, directed and co-ordinated by Dr. Tahir Maqbool, Dr. Faheem Hadi, Dr. Sehrish Razzaq, Sadia Naz, Dr. Saira Aftab, Dr. Sana Javaid Awan, Ayesha Nawaz, Farah Abid and Prof. Dr. Arif Malik. The manuscript was written by Dr. Tahir Maqbool and Dr. Faheem Hadi.

## Approval by Scientific Body

This study was approved by Departmental Scientific Review Committee of The University of Lahore.
